# Potential of APSIS-InSAR for measuring surface oscillations of tropical peatlands

**DOI:** 10.1371/journal.pone.0298939

**Published:** 2024-02-23

**Authors:** Martha J. Ledger, Andrew Sowter, Keith Morrison, Chris D. Evans, David J. Large, Ahmed Athab, David Gee, Chloe Brown, Sofie Sjögersten

**Affiliations:** 1 School of Biosciences, University of Nottingham, Sutton Bonington, Loughborough, United Kingdom; 2 School of Biological Sciences, Kadoorie Biological Sciences Building, The University of Hong Kong, Hong Kong SAR, China; 3 Terra Motion Limited, Ingenuity Centre, Nottingham, United Kingdom; 4 Department of Meteorology, University of Reading, Earley Gate, Reading, United Kingdom; 5 UK Centre for Ecology and Hydrology, Environment Centre Wales, Deiniol Road, Bangor, United Kingdom; 6 Faculty of Engineering, University of Nottingham, Nottingham, United Kingdom; 7 School of Geography, University of Nottingham, Nottingham, United Kingdom; Kanazawa University: Kanazawa Daigaku, JAPAN

## Abstract

Tropical peatland across Southeast Asia is drained extensively for production of pulpwood, palm oil and other food crops. Associated increases in peat decomposition have led to widespread subsidence, deterioration of peat condition and CO_2_ emissions. However, quantification of subsidence and peat condition from these processes is challenging due to the scale and inaccessibility of dense tropical peat swamp forests. The development of satellite interferometric synthetic aperture radar (InSAR) has the potential to solve this problem. The Advanced Pixel System using Intermittent Baseline Subset (APSIS, formerly ISBAS) modelling technique provides improved coverage across almost all land surfaces irrespective of ground cover, enabling derivation of a time series of tropical peatland surface oscillations across whole catchments. This study aimed to establish the extent to which APSIS-InSAR can monitor seasonal patterns of tropical peat surface oscillations at North Selangor Peat Swamp Forest, Peninsular Malaysia. Results showed that C-band SAR could penetrate the forest canopy over tropical peat swamp forests intermittently and was applicable to a range of land covers. Therefore the APSIS technique has the potential for monitoring peat surface oscillations under tropical forest canopy using regularly acquired C-band Sentinel-1 InSAR data, enabling continuous monitoring of tropical peatland surface motion at a spatial resolution of 20 m.

## 1. Introduction

Tropical peat swamp forests are globally significant terrestrial carbon sinks, storing an estimated 152–288 Gt C [1]. Such large carbon stocks have accumulated through waterlogging of the peat profile under conditions of restricted drainage, creating an anoxic environment whereby any organic biomass is unable to decompose, allowing for the accumulation of peat [2]. A large proportion of known tropical peatland resources are located in SE Asia, with an estimated coverage of 21 Mha [3] and carbon stocks of 68.9 Gt C [4].

Tropical peat swamp forests are a sensitive and unique ecosystem of interactions between above-ground biomass, peat and hydrology, with close coupling between the mean water table level and the height of the peat surface [5–7]. A change in any of these components can lead to a feedback loop of changes in the whole ecosystem [8, 9]. As a result, tropical peat swamp forests are highly sensitive to disturbance. In SE Asia, a surge in logging and agricultural development since the 1960s has led to widespread degradation and loss [10]. As of 2015, around 70% of the original extent of SE Asian peat swamp forest has been lost, with 20% converted to oil palm plantations alone [11]. The conversion process involves extensive deforestation, drainage and compaction of the peat for planting, resulting in widespread subsidence and changes in peat surface oscillation patterns due to changes in physical, biological and hydrological peat properties [12].

Subsidence and surface oscillations of tropical peat swamp forests are challenging to monitor on the ground. They are spatially and temporally complex processes whereby local disturbances can have system-wide ramifications [13]. As such, while some regional coherence in peatland surface motion has been observed [7] it remains difficult to extrapolate infrequent and localised field-based measurements to whole peatland systems. More practically, tropical peat swamp forests are spatially inaccessible environments that are costly to effectively monitor using field-based methods. Overall, the use of field-based measurements alone to monitor tropical peat swamp forest surface motion is unlikely to be achievable across the vast extent of all SE Asian peatlands.

The application of Interferometric Synthetic Aperture Radar (InSAR) could contextualise field-based measurements with improved spatial and temporal monitoring [14]. A range of different SAR wavelengths can provide information regarding different aspects of the tropical peat swamp forest environment. In general, for inundated vegetated areas (such as tropical peat swamp forests), the forest canopy or dry land will produce volume scattering and flooded areas will produce specular or double-bounce scattering [15]. The optimum system for detecting tropical peat surface oscillations in these environments is L-band due to the ability of this wavelength to penetrate the tropical peat swamp forest canopy consistently [16–18]. However, pioneering studies in the Amazon floodplain [19] and Louisiana swamp forests [20] have shown that C-band SAR is capable of detecting changes in water level in inundated forest environments with both emergent and established vegetation. Comparatively, tropical peat swamp forests exhibit similar environmental characteristics: widespread presence of surface water, adequate surface moisture (where water tables are below the peat surface) and similar density and height of forest stand and emergent vegetation. Therefore, it is valuable to investigate whether adequate coherence also exists over these similar environments.

To date, the Small BAseline Subset (SBAS) method [21] has been used successfully to derive long-term average surface velocities over tropical peatlands [18, 22] thanks to low temporal decorrelation, but limited precision of measurement with use of L-band SAR. Izumi et al. [23] and Zheng et al. [24] have shown effective results using variants of the SBAS method with C-band SAR to create annual averages for tropical peat subsidence rates. However, a key limitation of PSI and SBAS is that they only observe stable targets with low temporal decorrelation and are therefore limited to measuring areas with high phase stability and capturing long-term rates of surface deformation only, resulting in poorer coverage of vegetated environments relative to other land covers. The Advanced Pixel System using Intermittent Baseline Subset (APSIS, formerly ISBAS) method [25] relaxes this requirement and allows for more variation in coherence. When this is applied, measurements over all terrain types, including forests, becomes possible. The APSIS method has been able to detect long-term peat surface motion in temperate [26] and tropical peatlands [27], as well as seasonal surface motion characteristics in temperate environments [28–30] using C-band SAR, allowing for greater precision of measurements of absolute deformation [31]. Thanks to the European Space Agency’s Sentinel-1 mission launched in 2014, a long-term archive of C-band SAR images means that time series monitoring with sub-centimetre accuracy can now be achieved [32]. More comprehensive exploration of the use of this technique using C-band SAR over forested peatlands to produce seasonal surface motion time series is therefore required.

The aim of this paper is to validate, conceptually and quantitatively, the use of C-band InSAR for deriving a time series of surface oscillations over tropical peat swamp forest environments by addressing the following research questions:

1. How does C-band SAR interact with the tropical peat swamp forest medium?
2. To what extent is C-band SAR capable of measuring a time series of tropical peat swamp forest surface oscillations?

This study combines evidence from three investigative approaches: 1) use of SAR interferometry; 2) use of optimal mean SAR coherence (*coherence count*) from 2014–2020; and 3) use of LiDAR and optical datasets to describe tropical peat swamp forest stand and surface characteristics. The impact of above-ground biomass and peat surface characteristics on *coherence count* will be investigated to establish how C-band radar interacts with the medium of a tropical peat swamp forest.

## 2. Methods

### 2.1 Conceptual model: Radar imaging of tropical peat swamp forests

**Fig 1:**
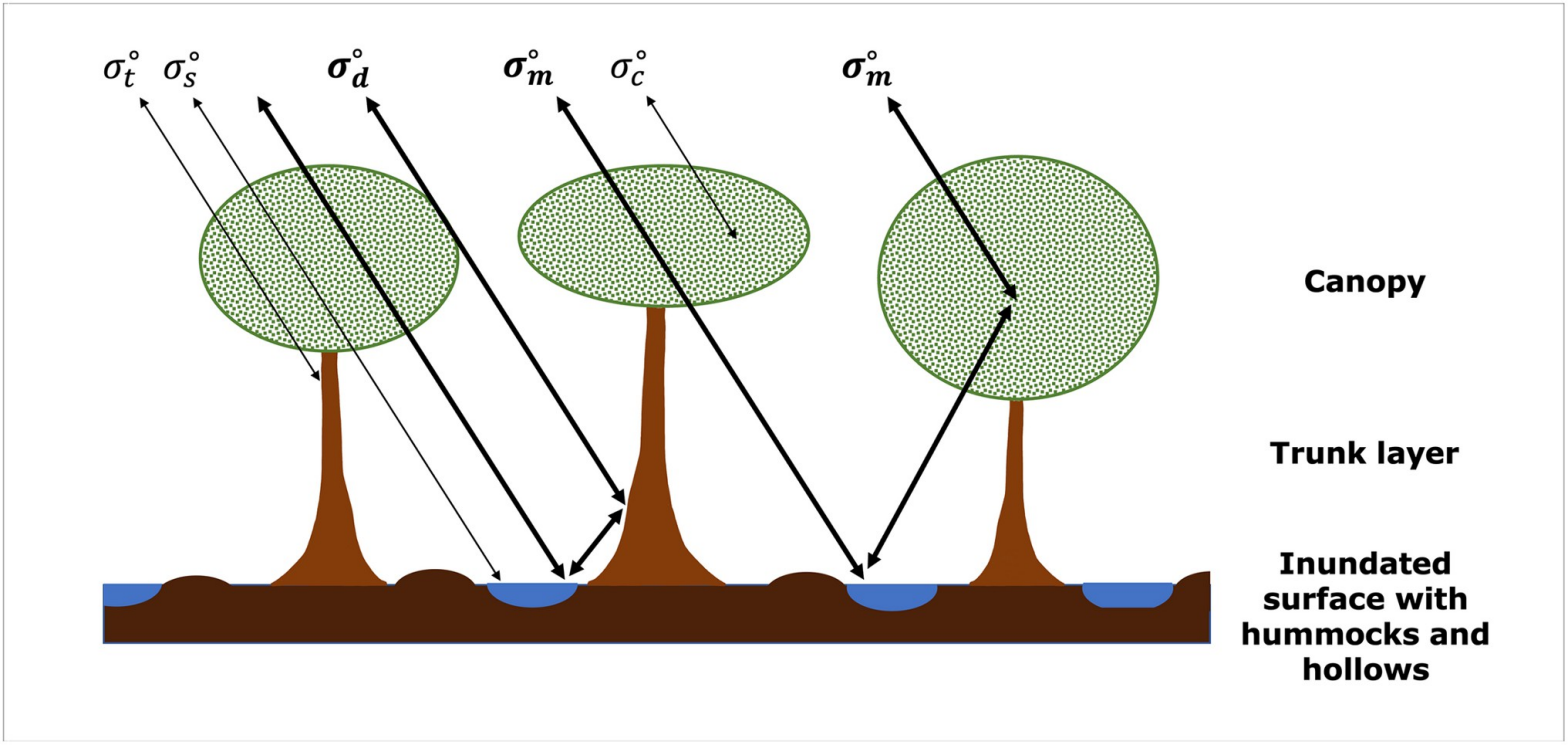
Diagram depicting sources of scattering from inundated forests (adapted from Kasischke and Bourgeau-Chavez [33]). Scattering mechanisms in bold represent scattering sources that result in interferometric fringes.

The radar scattering coefficient from a forest stand is a product of the interaction between direct scattering and signal attenuation within the forest stand, including canopy, trunk layer and the surface. This is conceptually summarised in Fig 1. This three-layer model was summarised by Kasischke and Bourgeau-Chavez [33] and can be expressed as:

$$\sigma^{\circ }=\;\sigma_{c}^{\circ }+\;\tau_{c}^{2}\tau_{t}^{2}\left(\sigma_{m}^{\circ }+\;\sigma_{t}^{\circ }\;+\;\sigma_{s}^{\circ }\;+\;\sigma_{d}^{\circ }\right)$$

$\sigma_{c}^{\circ }$ = *canopy volume scattering*;

$\tau_{c}^{2}$ = *transmissivity of crown layer*;

$\tau_{t}^{2}$ = *transmissivity of trunk layer*;

$\sigma_{m}^{\circ }$ = *multiple-bounce scattering between ground and canopy*;

$\sigma_{t}^{\circ }$ = *backscatter from trunk layer*;

$\sigma_{s}^{\circ }$ = *specular reflection from surface*;

$\sigma_{d}^{\circ }$ = *double-bounce between trunks and surface*

The ability to detect the tropical peat swamp forest surface is ultimately dependent on the transmissivity of the canopy and trunk layers and the reflectivity of the surface. This is determined by structural characteristics of the forest stand which are spatially heterogeneous across large forest expanses; variations in canopy closure, tree height, forest stand density, species, leaf shape, leaf size and leaf orientation in a secondary successional forest can lead to spatial differences in the ability to detect the peat swamp forest surface.

Without transmissivity of the canopy, the backscattering coefficient is dominated by volume scattering from within the canopy ($\sigma_{c}^{\circ }$). When the signal penetrates the canopy, it must interact with the trunk layer, where specular, double-bounce, multiple-bounce and trunk scattering ($\sigma_{s}^{\circ },\;\sigma_{d}^{\circ },\;\sigma_{m}^{\circ }$ and $\sigma_{t}^{\circ }$) occur. Double-bounce and multiple-bounce scattering ($\sigma_{d}^{\circ }$ and $\sigma_{m}^{\circ }$) in particular are complex phenomena that depend on the presence of corner reflectors to scatter the signal back towards the SAR antenna. Corner reflectors are objects that return a stable, persistent signal for the SAR antenna; in the tropical peat swamp forest environment, typical corner reflectors include stable tree trunks. Higher forest stand density can act as an enabler of these scattering mechanisms with more potential corner reflectors per unit area, as well as an inhibitor due to greater multiple-bounce potential, leading to increased signal attenuation. The ratio of canopy to trunk area also influences double-bounce and multiple-bounce scattering ($\sigma_{d}^{\circ }$ and $\sigma_{m}^{\circ }$) in the trunk layer of tropical peat swamp forests. Canopies with a greater proportion of canopy to trunk area will attenuate the double- or multiple-bounced signals more than canopies with a lower proportional volume [34]. Alternatively, forests with a greater proportion of trunk area will have less leaves or branches present to attenuate what remains of the signal after entrance through the canopy and scattering from the forest surface and trunks. Buttressed trees are particularly common in tropical peat swamp forest environments, which provide a greater area for corner reflectors than equivalent densities of forests with non-buttressed trees. Concentric zones of forest phasic communities exist on tropical peat swamps due to differing water table gradients [35]. Typically, stunted xeromorphic vegetation exists in the centre of the peat dome due to a low water table and a lack of nutrients from lateral flow inputs, and mixed swamp forest exists on the more fertile and waterlogged periphery of the dome [10]. This pattern is more complex at North Selangor, where extensive drainage and severe burn events have resulted in a fragmented forest stand, with a complex understory and extensive herbaceous vegetation dominating some areas of the reserve.

Surface characteristics are also very important in detecting scatter from the forest floor. Greater surface roughness, which is relative to the radar wavelength, leads to increased surface scattering ($\sigma_{s}^{\circ }$) and reduced double-bounce and multiple-bounce scattering ($\sigma_{d}^{\circ }$ and $\sigma_{m}^{\circ }$). Further, surface moisture content impacts the conductivity of the surface layer and therefore the reflection coefficient, whereby a moist ground layer (>10% GWC) has a higher dialectric constant, higher conductivity and therefore a high reflection coefficient. In conditions where surface roughness is constant, specular, double-bounce and multiple-bounce scattering mechanisms ($\sigma_{s}^{\circ },\;\sigma_{d}^{\circ }$ and $\sigma_{m}^{\circ }$) all increase. For forests with surface water, such as tropical peat swamp forests, the surface layer is assumed to be smooth, leading to specular reflection of the majority of energy reaching the surface. It is therefore assumed that there is no surface backscatter ($\sigma_{s}^{\circ }$) in the presence of standing water. As a result, any enhanced scattering that is detected from inundated forests results from double-bounce or multiple-bounce scattering ($\sigma_{d}^{\circ }$ or ${\;\sigma }_{m}^{\circ }$). This is because the signal is specularly reflected rather than diffused when interacting with the inundated surface.

The following metrics were used in this study to represent different structural characteristics of the forest stand, and therefore transmissivity of the canopy and trunk layers ($\tau_{c}^{2}\;$ and $\tau_{t}^{2}$) in the model: 1) Leaf Area Index (LAI [36]) ($\tau_{c}^{2})\;$ which is expressed as:

$$LAI=\frac{leaf\;area\;[m^{2}]}{ground\;area\;[m^{2}]}$$

**Fig 2:**
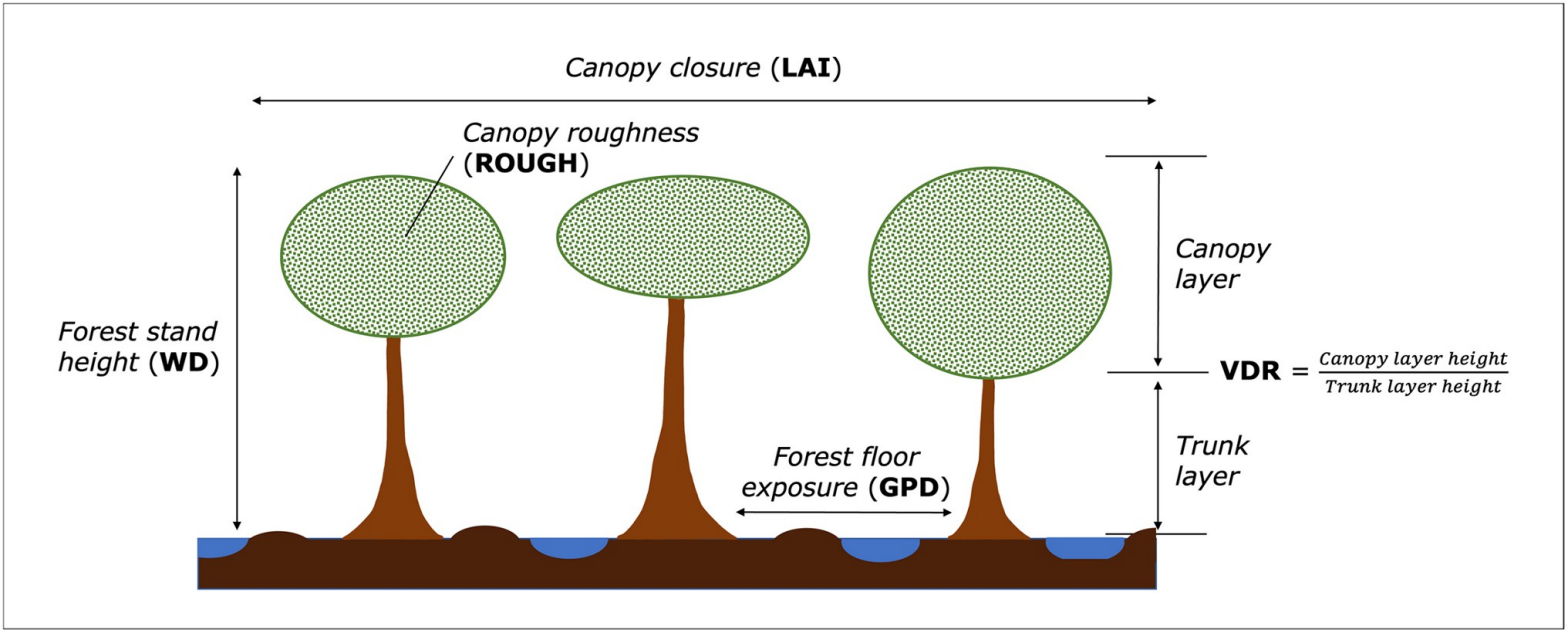
Diagram describing structural characteristics of the tropical peat swamp forest that impact scattering signals and associated metrics included in this study (adapted from Kasischke and Bourgeau-Chavez [33]).

A large LAI value represents greater leaf area cover and canopy closure; 2) ROUGH is a measure of canopy energy returns from LiDAR. It describes the roughness and therefore transmissivity of the crown layer ($\tau_{c}^{2})$. The lower the ROUGH value, the greater the scattering from the canopy volume; 3) Waveform Distance (WD) is a metric reflecting forest stand height, which impacts the amount of double-bounce and multiple-bounce backscattering ($\sigma_{d}^{\circ }$ and ${\;\sigma }_{m}^{\circ }$). A larger WD metric represents a taller forest stand; 4) Vertical Distribution Ratio (VDR) is an index of the vertical distribution of intercepted canopy components [37]. It is therefore a measure of the proportion of canopy in a forest stand, and the proportional volume of leaf and branch material through which the signal must penetrate ($\tau_{c}^{2}$). A high VDR means there is a greater proportion of canopy relative to trunk area; 5) Ground Point Density (GPD) is a measure of the exposure of the peat swamp forest floor. This is a function of canopy openness and forest stand density, and therefore determined by transmissivity of both the canopy and trunk layer ($\tau_{c}^{2}$ and $\tau_{t}^{2}).\;$ High GPD represents a more exposed tropical peat swamp surface. All measurements listed above are conceptually summarised in Fig 2.

Overall, double-bounce scattering is expected to be the primary explanation for any increased backscatter response from inundated forests [19, 20, 33]. However, variation in both canopy, trunk layer and surface characteristics across North Selangor present a challenge for detecting the tropical peat swamp forest surface on a frequent and spatially comprehensive basis. Nonetheless, evidence of intermittent coherence over time will support use of InSAR to create a time series of surface motion for the reserve, so long as there are enough interferometric pairs with coherent phase change.

### 2.2 Study site

**Fig 3:**
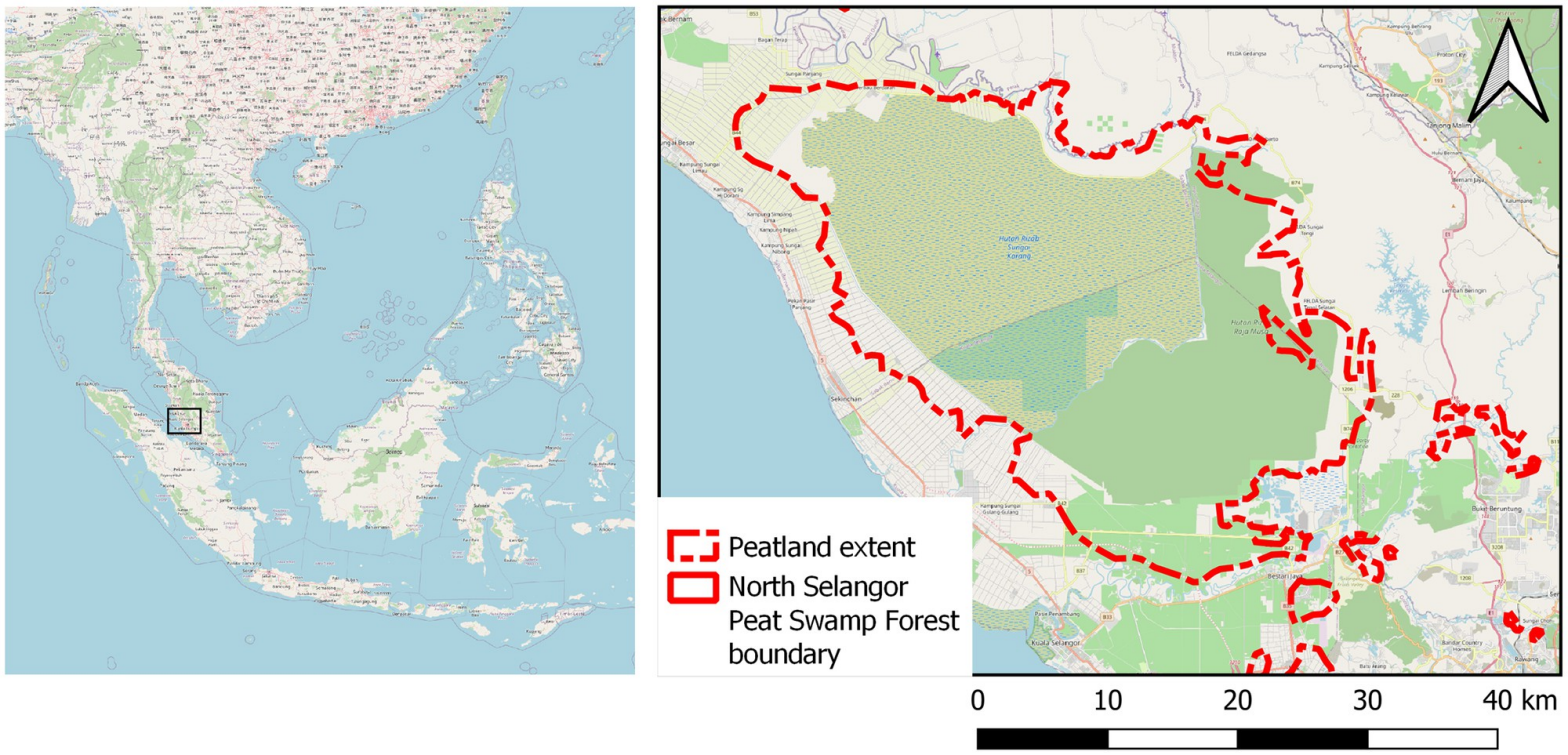
North Selangor reserve boundary and peatland extent located within Selangor, Peninsular Malaysia, SE Asia. Base map provided by OpenStreetMap^®^. OpenStreetMap^®^ is open data, licensed under the Open Data Commons Open Database License (ODbL) by the OpenStreetMap Foundation (OSMF). OpenStreetMap^®^ is made available under the Open Database License: http://opendatacommons.org/licenses/odbl/1.0/. Any rights in individual contents of the database are licensed under the Database Contents License: http://opendatacommons.org/licenses/dbcl/1.0/.

North Selangor Peat Swamp Forest (herein referred to as North Selangor) is located on Peninsular Malaysia (Fig 3), within which reserve status has been allocated. It covers an area of 814 km^2^, which is equivalent to the size of Singapore. Results from a forest management inventory conducted in 2000 state that the majority of the tropical peat swamp forest consists of a medium-height forest stand at mostly low- to medium-density of trees per unit area [38]. Trees remain in leaf all year round.

**Fig 4:**
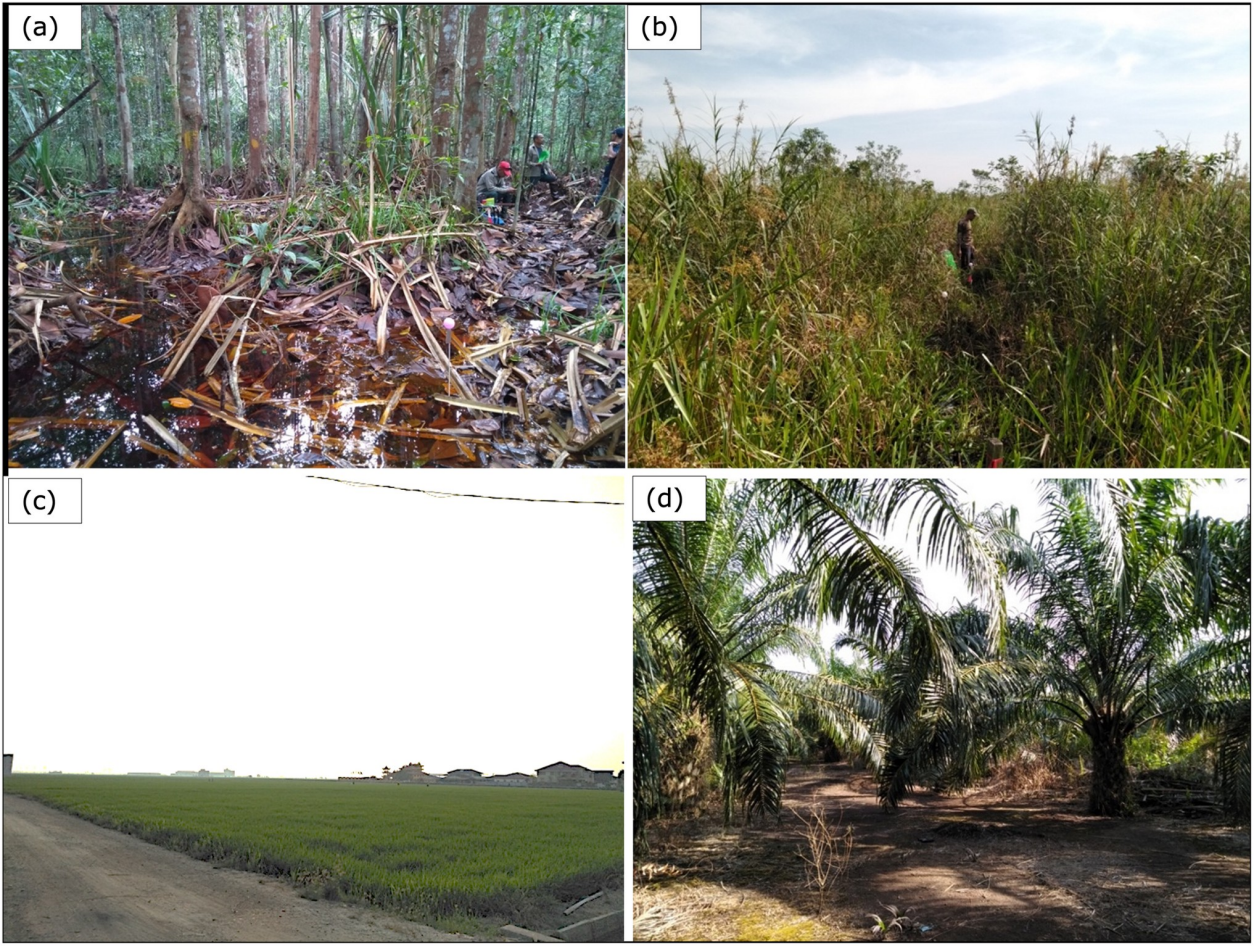
Photographs illustrating common land cover types and field conditions at north Selangor. (a) secondary forest, (b) burned peatland with dense grass and shrubs, (c) rice paddy agriculture and (d) oil palm plantation. (Photo credits: MJL).

Fig 4 illustrates North Selangor’s four main land cover classes at present: secondary forest within the majority of the reserve; burned grassland areas in the southeast of the reserve; rice paddies along the southwestern border; and oil palm plantations surrounding the remaining border. This variety of above-ground biomass type and density, agricultural practices and surface water presence makes North Selangor an ideal location for investigating the potential of C-band SAR for monitoring tropical peat swamp forest surface motion over a variety of land cover types.

### 2.3 Sentinel-1 data and processing

SAR amplitude and phase information was used to create interferograms and coherence maps for VV- and VH-polarisations. VV-polarisation data represents a signal that was emitted and received in the same plane, indicative of double-bounce scattering; VH-polarisation represents a signal that was sent in one plane and received in another, typical of volume and multiple-bounce scattering [39]. Production of interferograms and coherence maps enabled analysis of patterns of surface motion change across North Selangor, as well as an assessment of the quality of SAR phase across the reserve. Images were acquired in single-look complex interferometric wide swath mode, providing 5 m (range) by 20 m (azimuth) pixel resolution over a 250 km wide swath. Dates from the dry season were selected to reduce the risk of capturing rapid changes in surface level due to large rainfall inputs, which would lead to lower coherence. In this study we present four 12-day interferograms in VV- and VH-polarisations, produced from two C-band SAR image pairs exhibiting interferometric fringes dated 2/7/2017 and 17/7/2017, and 14/8/2018 and 28/8/2018.

Image pairs with small temporal and perpendicular baselines were selected to best maintain coherence; the temporal baselines were 12 days and the perpendicular baselines for the 2017 and 2018 pair were 1.7 m and 8 m, respectively. The small perpendicular baselines indicated that Sentinel-1 orbits are well-controlled and therefore suitable for tropical peat swamp forest interferometry. All images had the same track (track 70) and incidence angle (41.7° to 46.1°).

The Sentinel-1 data was processed using the *snaphu* toolbox, a phase unwrapping algorithm developed by Chen and Zebker [40], in ESA SNAP to create interferograms and coherence maps. Coherence is a measure of the phase coherence and is used to assess InSAR observation quality. It shows how similar each pixel is between the pair of images using a scale from 0 to 1, where higher values indicate higher similarity. Processing steps included the following: 1) coregistering the images using the S-1 Back Geocoding operator, 2) applying topographic phase removal based on the SRTM-3 DEM and 3) interferogram generation and coherence calculation. Coherence data were produced using a window of 7x2 pixels in the range and azimuth directions, respectively, for each interferogram. The Modified Goldstein filter [41] was applied to interferograms to increase the quality of existing fringes by enhancing the signal-to-noise ratio using Fast Fourier Transformation.

To extrapolate the coherence of the SAR interferometry over a longer time series (spanning both wet and dry seasons), the number of interferometric pairs with a mean coherence > 0.45 from 2014 to 2020 (*coherence count*) was calculated, with a maximum temporal baseline of 6 months. The *coherence count* is a measure of the consistency of coherence of point. 1520 interferograms were derived from 128 image combinations in VV-polarisation over 2028 days from 10/10/2014 to 29/04/2020 with a maximum span of 12 months between pairs. *Coherence count* scores therefore ranged from 0 to 1520 in line with the number of possible interferograms. The lower the *coherence count*, the fewer number of pairs included in the regression model to optimise the average coherence; therefore the coherence is more intermittent, and the poorer the quality of the InSAR data for creating a robust time series of measurements. In the case of this study, a *coherence count* threshold of 456 was applied as a measure of whether a time series could be adequately derived for a particular point; this threshold removed points where fewer than 30% of all interferograms met the mean coherence threshold of 0.45. Any points that did not meet this threshold would have resulted in sporadic and inaccurate time series of surface motion.

### 2.4 LiDAR data and processing

**Fig 5:**
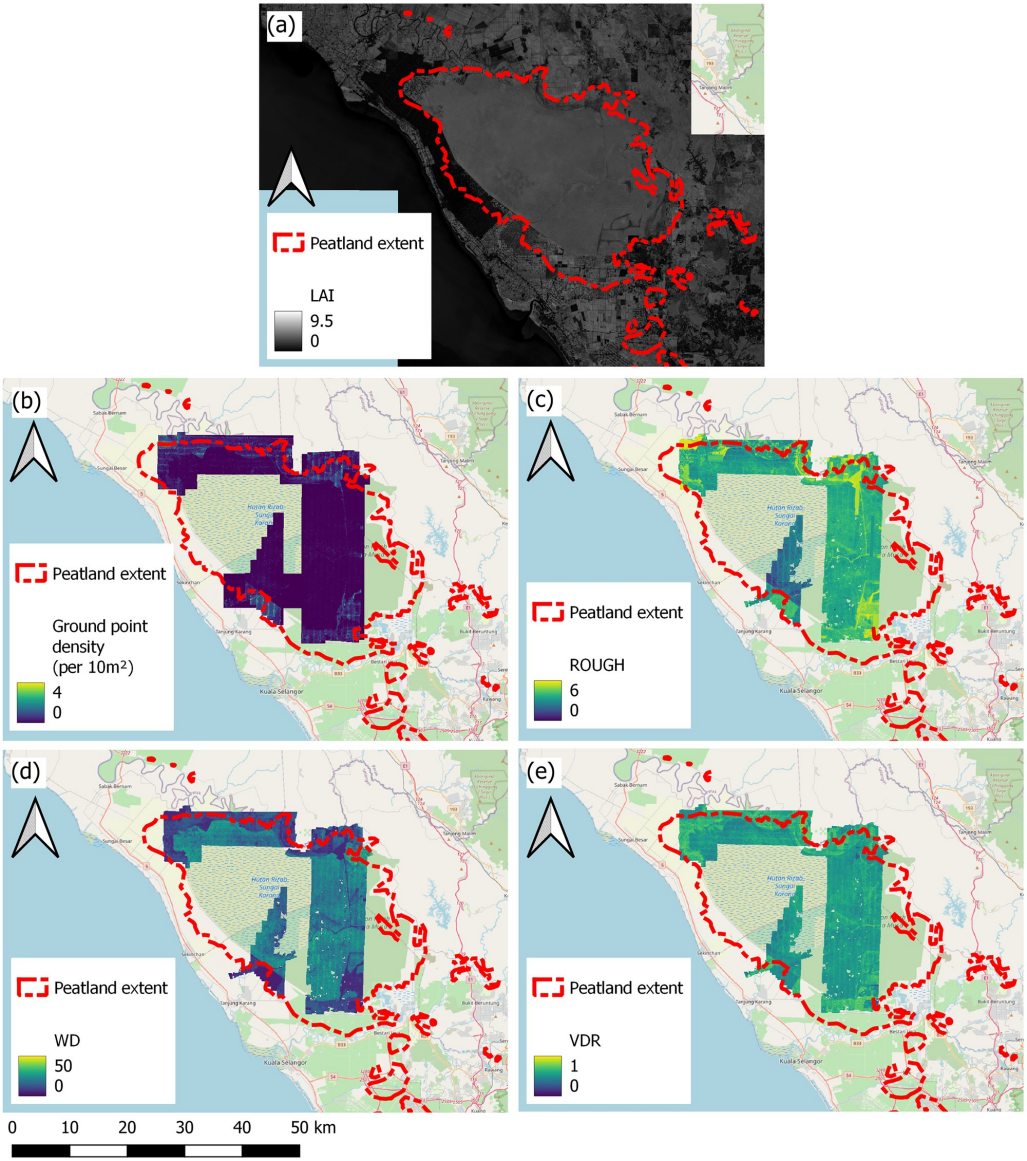
Maps presenting processed optical and LiDAR data, describing above-ground biomass structure and peat swamp surface characteristics, including (a) LAI (leaf area index per 20 m^2^); (b) LiDAR ground point density (ground point number per 10 m^2^); (c) ROUGH; (d) WD; (e) VDR. All LiDAR-derived datasets are limited to the LiDAR flight line coverage over North Selangor. Base map provided by OpenStreetMap^®^. OpenStreetMap^®^ is open data, licensed under the Open Data Commons Open Database License (ODbL) by the OpenStreetMap Foundation (OSMF). OpenStreetMap^®^ is made available under the Open Database License: http://opendatacommons.org/licenses/odbl/1.0/. Any rights in individual contents of the database are licensed under the Database Contents License: http://opendatacommons.org/licenses/dbcl/1.0/.

LiDAR data were included in the study to capture the physical characteristics of the forest stand and peat swamp surface. Airborne LiDAR data were collected by NERC ARF in 2014 using a Leica ALS50-II LiDAR system. Full waveform (FW) metrics are descriptors of the tropical peat swamp forest stand based on LiDAR returns, which were derived by Brown et al. [42]. FW metrics represent the following: the mid-point of the forest stand (HOME), canopy roughness (ROUGH; Fig 5c); the height of the forest stand (WD; Fig 5d); and the proportion of canopy layer to trunk layer (VDR; Fig 5e). To improve understanding of the peat surface characteristics at North Selangor, ground returns density data (Fig 5b) was derived from the LiDAR point cloud by filtering for ground points using ArcMap (ESRI).

### 2.5 Optical data and processing

LAI was used to provide a measure of canopy openness across North Selangor (Fig 5a). It was calculated from a cloud-free Sentinel-2 image dated 13/02/2018 using the Biophysical Processor toolbox on ESA SNAP.

### 2.6 Statistical analysis

Principal Component Analysis (PCA) was used to investigate the relationship between *coherence count* and peat swamp forest (LAI, WD, VDR, ROUGH) and surface characteristics (GPD). PCA is a descriptive statistical tool that requires no distributional assumptions. It is therefore a versatile, adaptive data analysis technique widely used on large and variable datasets [43], and applicable for exploring the relationship of C-band SAR with the tropical peat swamp environment.

## 3. Results

### 3.1 C-band SAR observation quality

**Fig 6:**
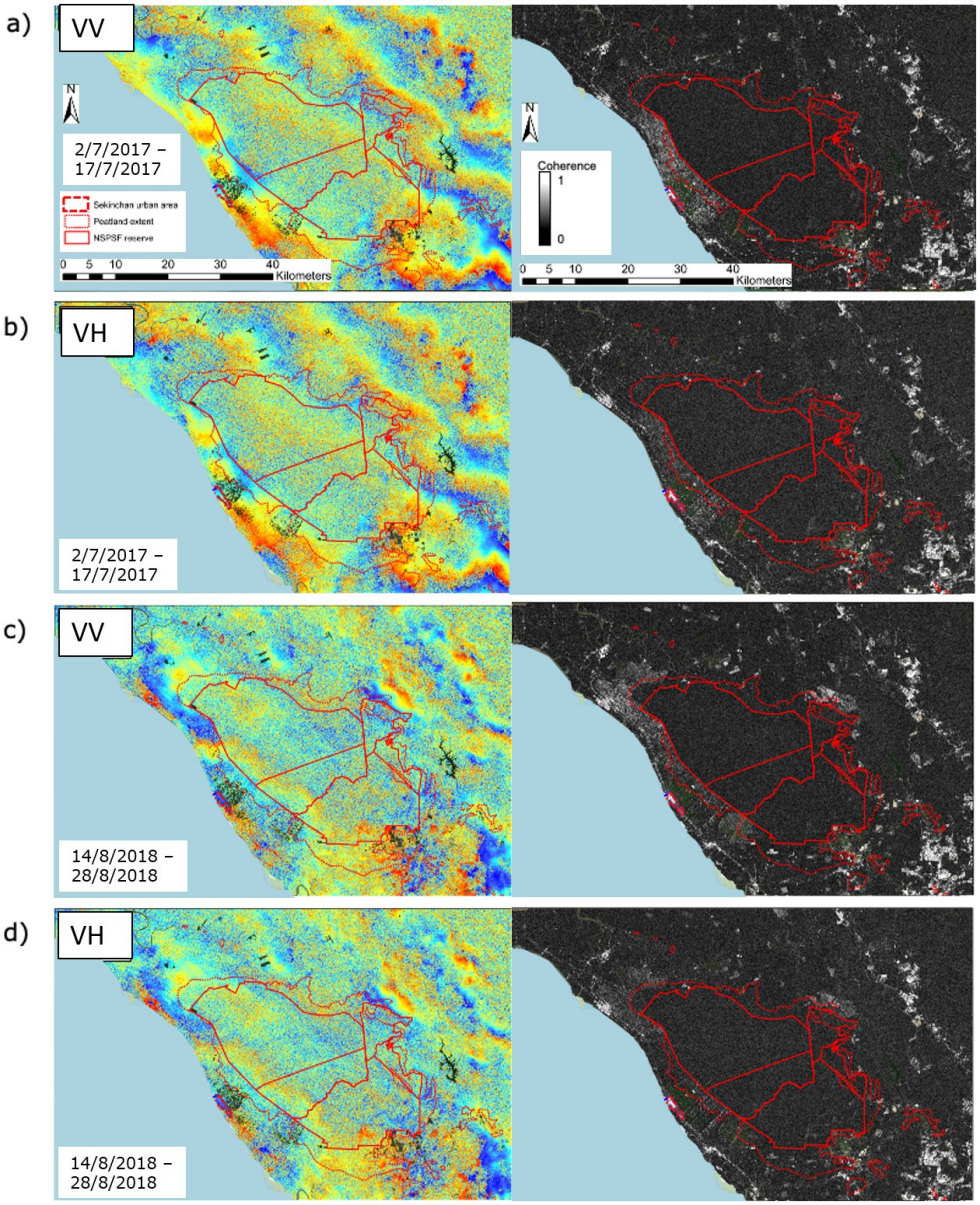
Sentinel-1 interferograms and coherence maps for North Selangor: a) VV polarisation from 2/7/2017 to 17/7/2017; b) VH polarisation from 2/7/2017 to 17/7/2017; c) VV polarisation from 14/8/2018 to 14/8/2018; d) the VH polarisation from 14/8/2018 to 14/8/2018. Base map provided by OpenStreetMap^®^. OpenStreetMap^®^ is open data, licensed under the Open Data Commons Open Database License (ODbL) by the OpenStreetMap Foundation (OSMF). OpenStreetMap^®^ is made available under the Open Database License: http://opendatacommons.org/licenses/odbl/1.0/. Any rights in individual contents of the database are licensed under the Database Contents License: http://opendatacommons.org/licenses/dbcl/1.0/.

Clear interferometric fringes are seen where coherent double- and multiple-bounce scattering dominates the InSAR signal [44, 45]. Alternatively, the presence of no fringes means that incoherent volume scattering constitutes most of the signal received. Phase interferograms for co- and cross-polarisations in Fig 6 showed that interferometric fringes were present, and that both polarisations exhibited similar fringe patterns. This indicated that phase was maintained across the majority of North Selangor for both polarisations.

The interferometric fringes exhibited a more diffusive pattern over North Selangor than neighbouring zones around the reserve, which is likely due to a higher proportion of volume scattering. Despite this, clear interferometric fringe presence across the peatland system indicated that a notable proportion of the C-band signal return must have come from double-bounce interaction between tree trunks and the saturated peat surface.

The coherence maps (Fig 6) showed that urban areas and rice paddies exhibited the highest coherence, with lower coherence present across the reserve where forest cover dominates. This was despite the presence of clear interferometric fringes within the reserve.

**Fig 7:**
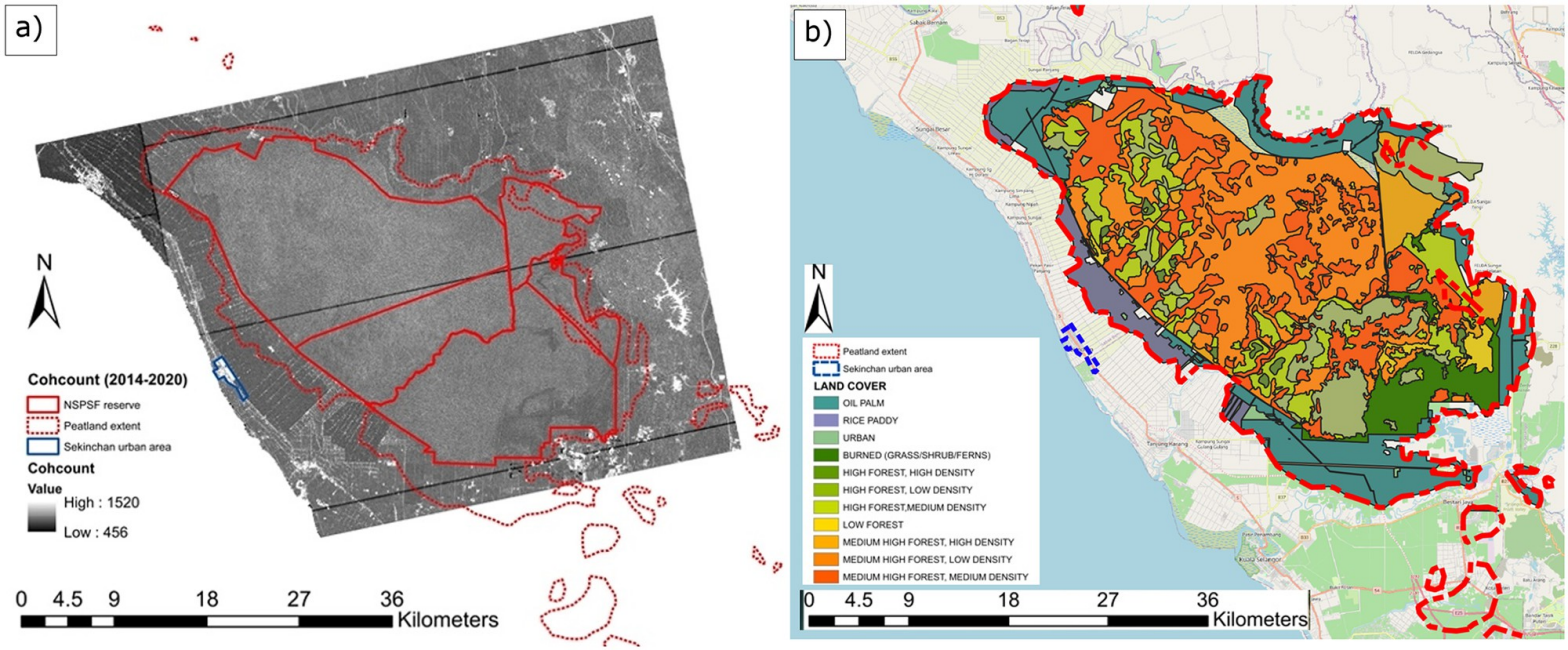
(a) Map of *coherence counts* (cohcount) of interferometric pairs at 20 m resolution between 10/10/2014 and 29/04/2020. The *coherence count* represents the number of interferometric pairs that are above the coherence threshold of 0.45 (maximum = 1520). (b) Map of land cover for North Selangor. Study of both maps show that *coherence counts* remained high over forestry within North Selangor relative to other land cover types. Base map provided by OpenStreetMap^®^. OpenStreetMap^®^ is open data, licensed under the Open Data Commons Open Database License (ODbL) by the OpenStreetMap Foundation (OSMF). OpenStreetMap^®^ is made available under the Open Database License: http://opendatacommons.org/licenses/odbl/1.0/. Any rights in individual contents of the database are licensed under the Database Contents License: http://opendatacommons.org/licenses/dbcl/1.0/.

To expand the significance of the presence of fringes in these two interferograms over a longer time frame, the number of interferometric pairs with an optimised mean coherence >0.45 (*coherence count*) for 2014–2020 was calculated for each pixel. Fig 7 showed a widespread moderate to high *coherence count* within North Selangor, particularly relative to grassland and rice paddy land cover, which had no forest cover, greater surface exposure and a smoother surface for the SAR signal to interact with (see WD, GPD and ROUGH in Fig 5).

### 3.2 Impact of peat swamp forest properties on *coherence count*

**Fig 8:**
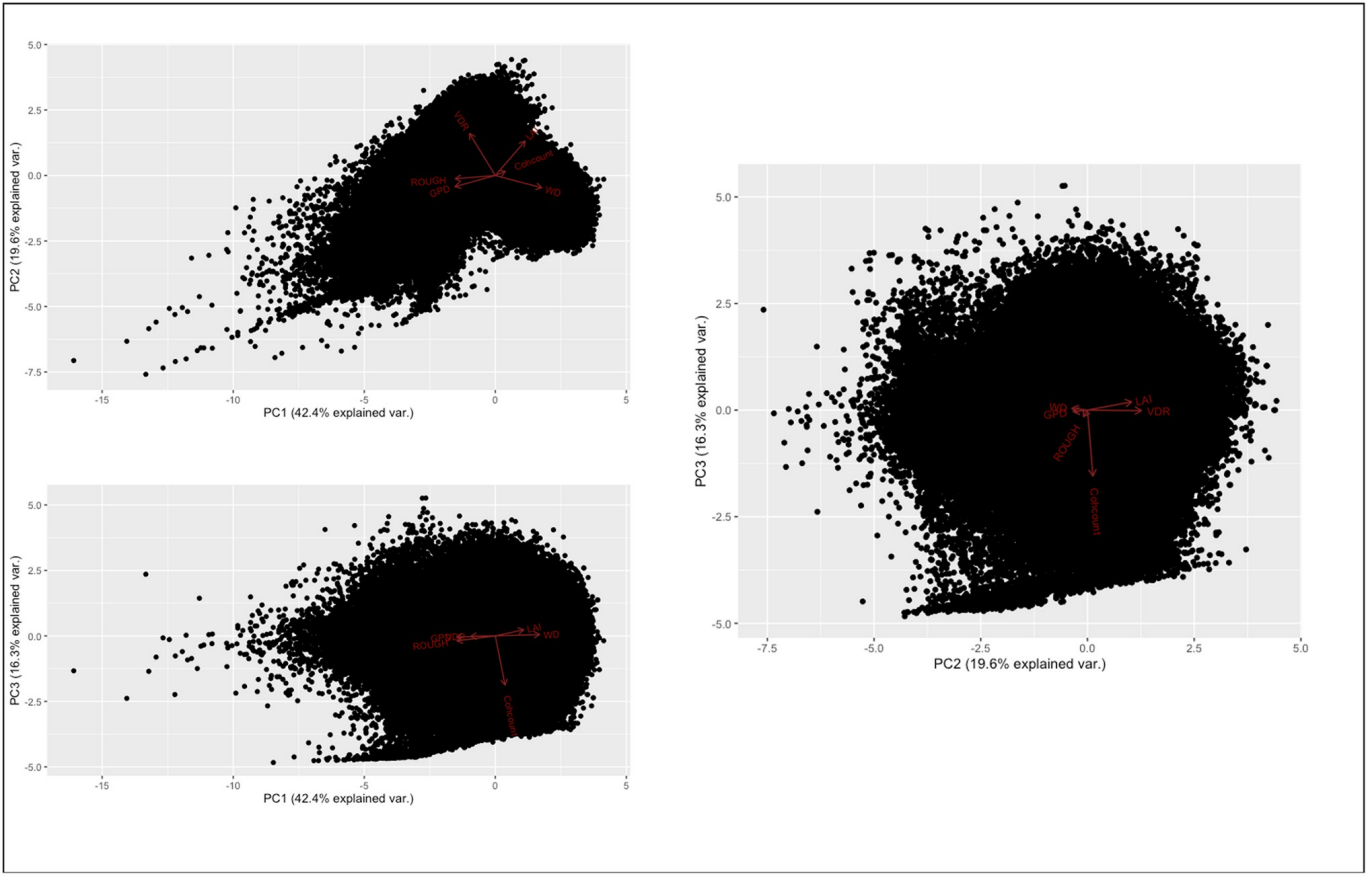
PCA visualising the relationship between *coherence count* (cohcount) values and metrics describing the canopy, trunk and surface layers in the model of the above-ground biomass stand: LAI (canopy openness), ROUGH (canopy roughness), WD (height of forest stand), VDR (ratio of canopy layer to trunk layer) and GPD (exposure of peat swamp forest surface). Plots represent all 2-dimensional combinations of the first three Principal Components: (a) PC1 and PC2, (b) PC1 and PC3, (c) PC2 and PC3. All three plots show that *coherence count* is orthogonal to all other variables.

Results from the investigation into the influence of different tropical peat swamp forest characteristics on *coherence count* data showed that the *coherence count* was not dictated by differences in tropical peat swamp forest characteristics. PCA showed that *coherence count* was orthogonal to the peat swamp forest medium metrics (LAI, WD, VDR, ROUGH and GPD) and above-ground biomass classification (Fig 8).

## 4. Discussion

### 4.1 C-band SAR interaction with the tropical peat swamp forest

This study suggested that C-band InSAR can indeed penetrate the forest canopy of tropical peat swamp forest environments, and that there is potential for monitoring changes in surface level in a challenging environment. Previously, it was assumed that C-band radar interacts mostly with the canopy, which is not a stable scatterer over time and produces poor InSAR results [34, 46]. However, the clearly defined fringes suggest that the phase information reflected C-band scatter interaction with the understory of the tropical peat swamp forest (i.e. trunks, branches and peat surface) between acquisitions and that double- and multiple-bounce scattering from the trunk layer is more important in these environments than commonly believed. Therefore, the results can rule out volume scattering as the only scattering mechanism in tropical peat swamp forest environments.

The two scattering mechanisms behind the coherence over North Selangor peat swamp forest are likely double-bounce and multiple-bounce. Studies from Richards et al. [47], Alsdorf et al. [19], Lu et al. [20] and Hong and Wdowinski [39] suggested that InSAR over wetlands maintains coherence thanks to the double-bounce effect, where the radar pulse scatters between the wetland surface and tree trunks. If clear fringes are found in the cross-polarisation, as was the case at North Selangor for C-band radar, then multiple-bounce is assumed to dominate the signal and this polarisation is indicative of surface level changes at this wavelength. It is unlikely that the signal would be as coherent if the overwhelmingly dominant scattering mechanism was volume scattering from the forest canopy [44, 45].

Wang et al. [34] and Kasischke and Bourgeau-Chavez [33] hypothesised that the ability of C-VV SAR to detect flooding beneath forests was dependent on structural characteristics of the forest, e.g. LAI, forest stand density, canopy closure, the vertical structure of the canopy and species composition. Townsend [48] found that forest structure and scattering at the trunk layer most strongly influenced double-bounce scattering of the C-VV signal in these environments. In particular, the larger the basal area (equivalent to forest stand density) and the taller the forest, the greater the amount of double-bounce scatter detected. Inundated successional forests were noted as particularly difficult to detect, which is characteristic of North Selangor. However, results from the interferograms produced in this study (Fig 6), and the derivation of *coherence counts* to represent average coherence over time (Fig 7a), showed that intermittent detection of inundation of tropical peat swamp forests was possible. Further, results showed that intermittent detection was possible across all forest types (Fig 7b), irrespective of canopy, trunk and surface characteristics, and was in fact higher on average compared to grass and rice paddy cover, which are more likely to produce specular reflection of the signal.

With a lack of relationship between *coherence counts* and forest stand characteristics, this study introduced the possibility that intermittent penetration of the canopy is random. To confirm this, further investigation is required using four approaches. Firstly, a study into the coherence of all pairs in the time series to see whether coherence (and therefore penetration through the canopy) is temporally random would be valuable; this would confirm that coherence is not determined by tropical peat swamp forest stand characteristics in North Selangor but by random intermittent penetration of the canopy. Secondly, it is assumed that the surface layer of the tropical peat swamp forest model is a constant coherent component of the forest. However, seasonality of precipitation in the low latitudes, as well as irrigation practices, means that the moisture of the surface layer varies throughout the year. This can affect the attenuation of the signal in the surface of the tropical peat profile for C-band SAR, as seen in south Florida wetlands [49]. Such seasonality also exists at North Selangor, therefore the peat surface cannot be considered a constant coherent component in this case. A ground-based C-band radar investigation experiment would be useful for confirming the assumptions of C-band radar’s inability to penetrate the tropical peat profile at current surface moisture levels at North Selangor [50]. Thirdly, more information on leaf size and phenology across North Selangor would also be valuable. Wang et al. [34] found that when leaf size was comparable to or larger than C-band wavelength, LAI had a large effect on the simulated C-band backscatter from flooded forests. Further investigation towards leaf size at North Selangor could establish if the poor relationship between *coherence counts* and LAI in this study is due to lower crown density and smaller leaf size at North Selangor, aiding canopy transmission across the reserve. Additionally, although phenology of tropical rainforests is expected to change very little [51], leaf production and shedding can be forced by extended dry periods, such as El Niño Southern Oscillation (ENSO) patterns. An understanding of the phenology of species at North Selangor would help to determine whether coherence is a function of phenology. Lastly, a polarimetric decomposition study would show the spatial distribution and prevalence of different scattering mechanisms across the North Selangor peatland extent. However, at present quad-polarisation coverage of North Selangor is poor, with only four images available from 2008 from the quad-polarisation-enabled RADARSAT C-band platform.

### 4.2 Capability of C-band SAR for measuring a time series of tropical peat swamp forest surface oscillations

A time series of surface motion can be created with only intermittent coherence from random canopy penetration using C-band InSAR. So long as enough interferometric pairs can detect the inundated surface, surface motion can be calculated. This has been possible over North Selangor, where the majority of the area had at least 456 pairs with an average coherence of >0.45. This meant that enough coherent interferometric pairs were available to create a time series of surface motion for 128 observations between 10/10/2014 and 29/04/2020 for the entire reserve. So far this has also been shown to be possible using an alternative ‘subset SBAS’ time series approach [23] and a ‘high coherence temporal subsets SBAS’ approach [24] over tropical peatland sites in Indonesia, although only by deriving annual averages per year.

This study established that in forested conditions, the tropical peat swamp surface contributed to the coherent signal at North Selangor, dependent on temporally random canopy penetration. However, it must be confirmed whether this model applies to other tropical peat swamp forest environments. Undisturbed tropical peat swamp forests characteristically have dense tree cover and high floral diversity [52]. They are controlled by hydrology, climate, underlying geology, nutrient availability and vegetation dynamics. Hydrology is an especially important control, especially for SAR observations, as it controls surface water presence, types of vegetation and rates of surface accumulation and decomposition. Seasonal flooding is widespread due to seasonal precipitation and/or river dynamics, where large and rapid surface level changes can occur, in the order of 10–20 cm [53, 54]. These magnitudes exceed the C-band wavelength (5.6 cm), which could lead to potential interferometric fringe ambiguities and an underestimation of surface motion, as documented by Umarhadi et al. [55]. On the other hand, high surface moisture levels all year round combined with an established forest stand mean that tropical peat swamp forests could exhibit sufficient intermittent coherence over the course of a longer period, as was the case at North Selangor.

However, in SE Asia, widespread logging has occurred since the 1960s, leading to the construction of networks of ditches which has resulted in extensive drainage and subsidence of many tropical peat swamp forests. As of 2015, Miettinen et al. [11] estimated that 70% of the original extent of SE Asian peat swamp forest had been lost, with 20% drained and converted to oil palm plantations. Comparatively, South and Central American and African peatlands remain relatively understudied compared to SE Asian peatlands [56]. However, it is largely agreed that tropical peatlands in these continents are anthropogenically disturbed to a much lesser degree, but that risk of degradation is rising in these areas with an increase in forestry and commercial agriculture, exploitation of the water resource and urbanisation [57–60]. Further, fire is an increasing risk to these environments with the exacerbation of climate change [61, 62]. Drainage, associated agricultural conversion and fire all typically result in the removal of forest cover and reduction in soil moisture, which have implications for SAR backscattering coherence in these disturbed environments in SE Asia. Absence of standing water will increase the roughness of the surface layer and therefore result in a more diffuse backscattering signal and lower coherence. Removal of trees will reduce the opportunity for double- and multiple-bounce backscatter and reduce the likelihood of coherence. As a result, tropical peat swamp forest systems overall are less likely to produce coherent pairs into the future.

Despite these anticipated changes in peat swamp forest environments, the planned continuation of current missions, recent advances in data availability and the launch of new missions mean that the APSIS time series method will be even more widely applicable for long-term seasonal time series analysis. Firstly, the anticipated launches of Sentinel-1C and Sentinel-1D will expand the C-band data acquisition period up to at least 2031, providing at least 17 years of regularly acquired data with global coverage [63]. This will produce the longest time series of freely available C-band data yet. Furthermore, the Japan Aerospace Exploration Agency (JAXA) ALOS-2 PALSAR-2 ScanSAR data with L-band wavelength at 14 day repeat pass has very recently been made publicly available, and the impending NASA (North American Space Agency) and ISRO (Indian Space Research Organisation) mission (known as NISAR) will be the first dual-frequency mission (L-band and S-band) to be launched. With acquisitions every 12 days, this mission will have great potential for subsidence monitoring, soil moisture estimation [64], and therefore forest and wetland carbon estimation [65]. After the planned 3-year lifespan, and potential follow-up missions, the NISAR mission, alongside the ALOS-2 data, present great potential for the production of a long-time series of freely available L-band data for seasonal tropical peat swamp forest subsidence monitoring. With the many benefits of L-band data for peatland subsidence monitoring explored previously [16–18], this mission also presents great potential for widespread and regular monitoring of tropical peatland systems with high coherence in the future. Overall, the scheduled provision of both L-band and C-band for tropical peat swamp forest surface oscillation monitoring means that complimentary analysis optimising both spatial coverage and accuracy of displacement rates could take place.

## 5. Conclusion

This proof-of-concept study has shown the potential for C-band Sentinel-1 radar to penetrate the forest canopy in leaf-on conditions over tropical peat swamp forests, and has shown that it is applicable to a wide range of land covers. It indicates that moderately dense tropical peat swamp forests enable double- and multiple-bounce returns for C-band radar in leaf-on conditions, providing the possibility that coherence over these environments is temporally random. With the sufficient time series length and frequency of acquisition provided by Sentinel-1, the APSIS-InSAR technique could characterise the spatial and temporal dynamics of tropical peat surface displacement on a regional scale, and with greater applicability in the future.
